# Immigrant and Swedish adolescents’ involvement in organized sports activities: an expectancy-value approach

**DOI:** 10.1186/s40359-021-00522-9

**Published:** 2021-01-23

**Authors:** Darun Jaf, Metin Özdemir, Therése Skoog

**Affiliations:** 1 Center for Lifespan Developmental Research (LEADER), School of Law, Psychology and Social Work, Örebro University, 701 82 Örebro, Sweden; 2 Department of Psychology, University of Gothenburg, PO Box 500, 405 30 Göteborg, Sweden

**Keywords:** Family socialization, Immigrant youth, Sports’ values, Organized sports, Expectancy-value model

## Abstract

**Background:**

Drawing on Eccles’ expectancy-value model, we investigated the associations between parents’ sports-related socialization behaviors in the family context, youth’s sports’ values, and youth’s involvement in organized sports activities in the Nordic countries. More specifically, we tested the mediating effect of youth’s sports’ values on the link between socialization of sports in the family setting and youth’s sports participation. Further, we examined whether any associations were moderated by youth’s immigrant background or gender.

**Methods:**

Immigrant and Nordic adolescents (*N* = 678), in 7th–8th grade, were followed over two consecutive years and responded to surveys during regular class hours.

**Results:**

Supporting Eccles’ model, we found that sports-related family co-activities significantly predicted youth’s prospective sports-related behaviors through youth’s sports’ values. The mediation process was robust across both Nordic and immigrant youth and adolescent girls and boys. Further, our results revealed that parents’ role modeling of sports activities was linked to both the amount of time youth currently spend on sports and their continuation in sports through youth’s sports’ values, although these associations were only significant for immigrant youth.

**Conclusions:**

Our findings offer insights into how participation in organized sports activities can be promoted among both immigrant and Nordic youth and among boys and girls. Most importantly, the findings may have valuable implications for researchers, policymakers and practitioners interested in promoting youth’s involvement in organized sports activities. This especially applies to immigrant youth, given that the literature consistently reports lower sports involvement among immigrant youth than their native counterparts.

## Background

To maintain an active and healthy life, participation in organized sports has been systematically promoted by governmental and other public agencies in Sweden [1, 2]. In line with the societal adoption of policies emphasizing active living and sports, around 70% of adults in the ages 25 to 65 are engaged in physical or organized sports activities [3]. Further, 58% of youth (12–18 years-old) take part in organized sports on a regular basis [4]. Despite high rates of participation across society more than one-third of youth do not take part in organized sports activities, with immigrants constituting one group that participates less [5–7]. Understanding the reasons for youth to adopt a physically active life by engaging in organized sports, and the reasons for continued engagement in such activities, is important for promoting sports participation among non-active groups. Adolescence is a developmental period marked by increased incidence of mental health problems, such as anxiety and mood disorders [8, 9], and of risky behaviors, including substance use and norm-breaking [10, 11]. The benefits of organized sports are not limited to offering youth a physically active and healthy lifestyle, but, also offer opportunities to cope with challenging periods by participating in a developmental setting that often is linked to youth’s positive adjustment across the behavioral and psychological domains [12–14]. Thus, in light of the expectancy-value model [15], which emphasizes the socializing role of parents and family in youth’s choice of activity participation, the aim of the current study is to examine the links between immigrant and native Nordic youth’s sports-related experiences in a family context, their subjective valuations of sports activities, and their involvement in organized sports.

## Eccles’ expectancy-value model

In her model, Eccles highlights two distinct, yet closely related, aspects of behavior that are of particular relevance to questions concerning youth’s choice of activity participation: youth’s task-related values, and socialization processes within the family environment [15–17]. Specifically, it is argued that socialization processes play a central role in shaping youth’s values with regard to activities, which in turn influence youth’s enrolment in and continuation with a specific activity (e.g., sports). These ideas have received support across a range of longitudinal studies [18–20].

### Youth’s task-related values

Within the expectancy-value model, task-related values are defined in terms of how a given task can fulfill different needs or desires in the individual [15, 16, 21]. For example, youth might value and place importance on a given activity: because of the enjoyment they get from participating in that activity (i.e., intrinsic value); because participation in that activity is important and useful for youth’s future goals (i.e., utility value or usefulness of an activity); or because participation in that activity allow youth to confirm and express important aspects of themselves (i.e., attainment values). Task-related values are proposed, and demonstrated in empirical studies, to be one of the most direct determinants of youth’s enrolment in and persistence with a given activity [15, 22–24].

Generally, scholars have either combined the different dimensions of task-related values (attainment value, intrinsic value, and utility value) into a single measure [18, 19] or used some of the dimensions separately [22–24]. However, less attention has been paid to the unique role played by attainment value in youth’s enrolment in and persistence with organized sports. Given the importance of attainment values to an individual’s perceived identity within any domain [17, 25], such values might have particular implications for youth’s choices of activity participation and continuation. Hence, in the present study, we mainly focus on youth’s sports-related attainment values.

### Socialization of sports within the family context

In the expectancy-value model, the family environment has an important role to play in youth’s interest and participation in extracurricular activities like organized sports [15, 25]. It is argued that parents, as the main socializers in the family context, can, through various behaviors, offer encouragement and experiences related to a specific activity. In turn, parents can influence and shape their youth’s task-related values. For example, as role models (e.g., own engagement in activities) parents can implicitly communicate their own values and preferences by engaging in certain activities (e.g., sports) rather than others [15, 26]. Based on observational-learning processes [27], Eccles [15] argued that, by observing their parents’ engagement in certain activities, youth may adopt behaviors and motivational beliefs like their parents’. Alternatively, parents can adopt more explicit behaviors that provide their children with activity-related experiences inside or outside the home [20, 28]. Specifically, by spending time together with their children (e.g., watching sports at home, or taking their children to a sports event at an arena) parents can transmit the values that are attached to an activity within the family [29, 30]. Taken together, it is argued that these (implicit and explicit) socialization behaviors may influence youth’s activity values, which, over time, will be integrated into youth’s own self-systems [26]. In turn, these processes will play a key role in youth’s choices regarding activity enrolment and continuation [28, 29, 31]. The link between family sports socialization and youth’s sports’ values has received support in several longitudinal studies [18, 19, 30]. In sum, the family context is an important determinant of youth’s task-related values. In the present study, we focus on both implicit (e.g., role-modeling) and explicit (e.g., watching sports at home or at a sports arena) socialization behaviors.

### Gender of the child

Eccles and colleagues [15, 31] have also argued that parents hold different expectations and beliefs about boy’s and girl’s competencies across extracurricular activities (e.g., sports and music). For example, within the sports domain, parents rate sports activities as more important for boys than girls, and boys’ sports abilities as higher than girls’ [19, 31]. In the music domain, however, parents show the opposite gender-related beliefs and behaviors, i.e., in favor of girls [19, 20]. As a result of these gender-stereotypic beliefs, it is suggested that parents use different socialization behaviors according to the child’s gender and type of activity (e.g., sports or music), which, in turn, contribute to gender differences in youth’s values and choices of activity involvement [15, 20]. Empirical research has demonstrated that both mothers and fathers socialize sports activities more for boys than they do for girls [18, 19, 30, 31]. For example, parents are likely to buy sports-related equipment, to encourage participation in sports, and to spend time on sports activities more for their sons than for their daughters [30]. Based on the perceptual bias that sports are more suited for boys than girls, we expected differences between boys’ and girl’s perceived socialization of sports in the family setting. In addition, we investigate whether the associations between family sports’ socialization behaviors and youth’s sports’ values are moderated by gender of the adolescents.

### The present study

Drawing on propositions from the expectancy-value model [15], the primary goal of the present study was to examine the roles of youth’s sports-related experiences in the family context and youth’s attainment values in their involvement in and continuation with organized sports. Scholars have generally examined the associations between family-level socialization constructs and youth characteristics as unidirectional influences going from the family to the child. Nevertheless, proponents of the expectancy-value model have also emphasized the possibility of reciprocal influences between the two distinct components. For example, parents may respond to their children’s needs and interests by socializing certain activities that accord with the children’s preferences. In a 12-year longitudinal study [32], it was found in cross-lagged analyses that children’s beliefs and behaviors did not influence their parents’ beliefs or socialization behaviors. Instead, the findings support the proposition that socialization behaviors in the family setting (e.g., role modeling, co-activity, encouragement) influence and shape children’s beliefs (e.g., subjective values regarding a specific activity) and behaviors (e.g., involvement in a specific activity). In line with these arguments, we examined the mediating role of youth’s attainment values in the association between socialization of sports in the family context and youth’s organized sports participation and continuation over time.

A secondary goal of the present study was to examine the moderating effect of youth’s immigrant background and gender. Little is known about whether propositions from the expectancy-value model apply similarly to immigrant families. The few existing findings based on research conducted in Denmark, a Scandinavian country very similar to Sweden in terms of both overall sports policy [1] and the diversity of immigrant inhabitants [33], revealed that immigrant parents are less likely to participate in sports than their native Danish counterparts [5]. There are similar findings for Sweden, where immigrant parents participate in physical and sports activities to a lesser extent than their Swedish counterparts [3]. Taken together, the findings suggest that, compared to their Nordic peers, fewer immigrant youth have a family context that socializes them into the development of sports-related values and engagement in sports activities. Hence, research from both Denmark [5, 33] and Sweden [4, 7] shows that immigrant youth report less sports engagement than their native peers. Nevertheless, immigrant parents who are engaged in sports or physical activities may still have similar socializing effects on their youth’s sports’ values and sports involvement. Findings from both empirical [5] and qualitative [34, 35] studies support this idea. For example, in a cross-sectional study of children’s participation in organized sports activities [5], the authors found that children of immigrant background reported significantly lower involvement than their Danish counterparts. However, subsequent analysis revealed that parent’s past and current involvement in sports activities explained most of the association between youth’s immigrant background and their involvement in organized sports. Taken together, these reports indicate that, like those of their native counterparts, immigrant parents’ socialization behaviors may play an important role in their children’s participation in organized activities. In line with these arguments, we examined whether the associations between youth’s sports-related experiences in the family context and youth’s attainment values, on the one hand, and their involvement and continuation in organized sports activities, on the other, are moderated by youth’s background (native or immigrant). Our findings may have important implications for practitioners and policymakers interested in developing parent-focused strategies to increase both immigrant and Nordic youth’s participation in and continuation with organized sports. Second, and most importantly, the findings may offer valuable insight into whether similar parent-focused strategies could be used to promote immigrant and Nordic youth’s involvement in structured after-school activities, such as organized sport.

Based on arguments from Eccles model [7] and previous research [19, 30], we expected to find mean-level differences between boys’ and girls’ reports of sports socialization within the family setting, subjective sports’ values, and participation in sports activities. Further, we hypothesized that any associations found between youth’s sports-related experiences in the family context and youth’s attainment values, on the one hand, and their participation in and continuation with organized sports, on the other, would be moderated by youth’s gender.

## Method

The data were from the Youth and Sports (YeS) project, a three-year longitudinal study with the overall aim of understanding how children and early adolescents who are involved, or not, in organized leisure activities (e.g., sports), develop over time. The data were collected from public-sector schools in a medium-sized city in central Sweden. The schools were selected from different neighborhoods to match the socio-demographic characteristics of the city. In terms of its unemployment rate, the city (7%) was the same as the national average (7%) [36]. It was also close to the national average in terms of annual income (303 300 Swedish kronor/person compared to the national average of 300 000 Swedish kronor/person) and number of immigrants born outside Sweden (22% compared to the national average of 18.64%).

In the present study the data were collected from early adolescents across 10 schools. The target group was 812 students in grade 7, 83% of whom (*N* = 678) were present during collection and took part in the study during the first year (46% girls, *M*_age_ = 14.90 and *SD*_age_ = 0.39). Of the first-year participants, 83% responded to the survey again in grade 8 (*N* = 563 44% girls, *M*_age_ = 15.90 and *SD*_age_ = 0.38). The analytic sample for the present study comprised adolescents who took part in the survey at the first (T1, *n* = 620) and second (T2, *n* = 563) years of the YeS project. At T1, 4% of parents refused to allow their children to participate, and at T2, 6%. A small proportion of the youth did not take part in the survey due to sickness during the first (*n* = 6) and second (*n* = 7) years, and due to truancy or for other (unknown) reason during the first (6%) and second (7%) years. Most of the participating youth were from intact families (63%), with employed parents (77% for mothers, and 89% for fathers), and 83% perceived their financial situation to be just as good as or better than their classmates.

Approximately one-third (29%) of the students or their parents were born outside a Nordic country (Sweden, Denmark, Finland, or Norway). Most of the participants spoke Swedish at home (70%), while about a third (30%) spoke a foreign language or the combination of a foreign language and Swedish at home. The parents of the immigrant youth had migrated from 48 different countries on different continents, including Asia, Africa, South America, and Europe. The most common part of the world from which the immigrants came was the Middle East (37%), including Iraq and Syria. These numbers are also reflected in national data where immigrants, born in a country other than Sweden, represent 19% of the total population in Sweden [37], and most immigrant families have come from Syria, Finland, and Iraq.

### Procedure

Parents received an information letter by regular post with a description of the project and were asked to return a postcard in a pre-paid envelope if they wanted to refuse the participation of their offspring in the study. Data collection from the youth took place during two regular class hours (approximately 90 min) and was administered by trained research assistants. Before the administration of the survey questions, research assistants presented a thorough description of the project, a statement about the voluntary nature of participation and confidentiality of information, and an assurance that participants could quit the study whenever they wanted. A few students with language difficulties received help from research assistants in the students’ first languages. The study was approved by the regional Ethics Review Board in Uppsala (Dnr: 2015/330).

## Measures

### Organized sports activities

In line with the literature on dimensions of youth’s involvement in organized activities [38], such as sports, we used three different measures of youth’s sports involvement: participation in organized sports, intensity of sports participation, and continuation with organized sports activities.

#### Participation in organized sports activities

The most common approach to measuring involvement in organized sports activities is to ask participants a yes-or-no question about whether they are involved in sports [18, 39, 40]. Consistent with the literature, we measured youth’s participation in organized sports activities using one dichotomous item “Are you involved in after-school sports activities, for example, soccer, ice-hockey, horse riding, athletics, running?” Youth who were involved in sports activities were coded as 1, the others as 0.

#### Intensity of sports participation

An alternative way of measuring sports participation is to tap into the amount of time youth spend on sports activities. This approach offers a better understanding of the extent to which youth are engaged in sports. In line with previous research [41, 42], we created an intensity-of-sports-participation index based on responses to two questions. The first item, “How many days per week do you usually practice?” was on an 8-point scale ranging from 0 (*No days*) to 7 (7 days a week). The second item, “On average, how many hours do you practice on each occasion?” concerned the duration of an ordinary practice session and was on a 5-point scale ranging from 0 (*less than 1 h*) to 5 (*more than 4 h*). To estimate the number of hours youth spent on sports activities, we multiplied the responses to these two questions, which resulted in a score ranging from 0 to15, reflecting the degree (i.e., intensity) of youth’s engagement in organized sports. For example, participants who engaged in sports activities 3 times per week and had a practice session that lasted for 2 h received an intensity score of 6. The maximum score was set at 15, which encompassed the scores of 90*%* of the youth. The scores of a small proportion of the youth, 7*%* at T1 and 10*%* at T2, were higher than 15 but were recoded as 15.

#### Continuation with sports activities

In line with the literature on duration and consistency of youth’s sports participation [40, 43], we measured youth’s persistence in sports activities as continued participation over time. We asked the same question about participation in sports activities at both T1 and T2: “Are you involved in after-school sports activities, for example, soccer, ice-hockey, horse riding, athletics, running?” We identified the youth who were involved in sports activities at T1 and who continued to be involved at T2. Youth who persisted with sports activities were coded as 1, and youth who were involved in sports activities at T1 but were no longer involved at T2 (i.e., who had dropped-out of sports) were coded as 0. Those who were not involved in organized sports at T1 were excluded from the analyses concerning continuation with organized sports activities.

### Sports-related attainment values

Following Eccles’ expectancy-value model [15, 22] we measured youth’s sports-related attainment values in relation to sports activities using four items. Examples include “Sport is the most important part of my life”, “I spend more time thinking about sport than anything else”. The items were on a 5-point scale ranging from 1 (*Disagree*) to 5 (*Agree*). Inter-item reliability was 0.89. Similar scales have been used previously to assess youth’s attainment values [15, 19].

### Sports-related socialization behaviors in the family context

Drawing on the expectancy-value model [15, 28, 31], we used two different measures to tap into youth’s perceptions of sports-related socialization behaviors in the family context: role-modeling behavior and sports-related family co-activities.

#### Role modeling

Role modeling was measured using two items. Initially, participants were given a dichotomous item to report on whether their parents were engaged in physical activities after work or at weekends: “Do your parents exercise after their work or at weekends?” Youth with parents involved in physical activities were coded as 1, and others as 0. Participants were also asked to report on how often their parents engaged in physical activities after work or at weekends “How often do your parents engage in sports or physical activities?” on a 4-point scale ranging from 1 (*almost every day*), 2 (*3–4 times*), 3 (*1–2 times*), and 4 (*less often*). The responses were reverse-coded so that higher values refer to more frequent engagement. We combined these two items into a single measure of parents’ role modeling of sports activities on a 5-point scale. Parents who were not involved in physical activities were coded as 0, and parents who were engaged in physical activities were scored between 1 (*less often*) and 4 (*almost every day*). These items were developed as part of the YeS project, following theoretical guidelines [15, 28, 31], and the practices of other scholars using similar items to measure parent’s role modeling of physical and sports activities [5, 19, 20].

#### Sports-related family co-activities

We assessed sports-related family co-activities using two questions. Specifically, we asked about the extent to which youth were engaged in sports-related activities with their family: “How often do you watch sports on TV with your family members?”, and “How often do you watch sports at sports arenas/stadiums with your family members?”. The items were on a 5-point scale ranging from 1 (*never*) to 5 (*twice or more than twice a week*). The correlation between the two items was *r* (641) = 0.44, *p* < 0.001. We used the mean of the responses to these two items as a measure of sports-related family co-activities. These items were developed as part of the YeS project, following theoretical guidelines [15, 28, 31], and the practices of other scholars using similar items to measure sports-related family co-activities [19, 20, 28].

### Socio-demographic characteristics

#### Immigrant background

Youth were asked about the birthplace of their parents. Using parent’s birthplace to identify youth’s immigrant background is a common approach in the literature [44–46]. This approach allowed us to identify both first-generation (born abroad) and second-generation (born in Sweden to foreign-born parents) immigrant youth. Participants with both of their parents born outside a Nordic country were regarded as immigrant youth. Participants with both of their parents born in a Nordic country (Sweden, Norway, Finland, or Denmark) were regarded as Nordic. A small group of adolescents (7%) had only one parent born outside the Nordic countries. This group was more similar to the Nordic youth than the immigrant youth on all the study variables. Thus, they were regarded as Nordic youth. Immigrant youth were coded as 1, and Nordic youth as 0.

#### Family socio-economic status

Participants also reported on their gender and age as part of the demographic questionnaire. In addition, we measured family socio-economic status (SES) based on mothers’ (“Does your mother have a job?”) and fathers’ (“Does your father have a job?”) employment status using two separate items coded as 0 (*No*) and 1 (*Yes)*. We then combined the two items: 0 = *parents are not working*, 1 = *one of the parents is working*”, and 2 = *both of the parents are working*. Family employment status is regarded as an adequate proxy for family SES [47].

#### Attrition and missing data analysis

Of the 620 adolescents in the initial analytic sample, 91% (*n* = 563) remained at T2. We performed logistic regression analyses to investigate whether the study variables were systematically related to attrition. Specifically, we regressed attrition (dropout = 1, retention = 0) on the demographic characteristics of the adolescents (i.e., age, gender, immigrant status, and parents’ employment status) and all the other study variables (i.e., participation in organized sports, youth’s sports’ values, parents’ role-modeling behaviors, and sports-related family co-activities). The results showed that none of the demographic characteristic or the study variables were related to attrition (Nagelkerke *R*^2^ = 0.03). Further, we inspected missing information within each wave across the study variables. Missing information ranged between 0.5 and 7%, with the exceptions of immigrant background (8%) and parents’ employment status (11%); the average was 2.18%. Missing values were estimated using the EM algorithm [48], which has been shown to produce relatively unbiased estimates compared to traditional missing data-treatment methods, such as listwise deletion, pairwise deletion, or mean imputation [49].

## Data analysis

Initially, we inspected the bivariate associations to see if the variables were associated with each other in the expected directions. To address the overall aim of the study we adopted an analytical strategy designed to test mediation and moderated mediation processes [50–52] using the PROCESS program [50] in SPSS. For the mediation processes, we examined whether the link between a predictor variable (e.g.., the socialization of sports in the family setting) and a dependent variable (e.g., a sports-participation dimension) was explained by a third intermediary/mediating variable (e.g., a youth’s attainment value). For the moderated mediation processes, we followed Hayes [51, 53] recommendations to examine if an indirect path (e.g.., family sports-socialization behaviors, through youth’s attainment values, to the sports-participation dimensions) was moderated by youth’s immigrant background or gender. The estimation of a moderated mediation in PROCESS macro, also called *index of moderated mediation* by Hayes, 2015, is computed based on these equations:

1 $$M = iM + a1X + a2W + a3XW + eM$$

2 $$Y = iY + c^{\prime}X + bM + eY$$

3 $$\omega = a1b + a3bW$$

The first equation includes coefficients for the predictors of **M**. For example, the coefficient of the predicting variable (*a1X*), the moderator (*a2W*), and the joint effect of the main predictor and the moderator (product of a1X*a2W = *a3XW*) on **M.** The second equation includes coefficients for the predictors of the outcome variable (**Y**). For example, the coefficient of the predicting variable (*c’X*) and the mediator (*bM*). The third equation includes the estimation of the indirect effect as a function of the moderator. For example, the product of the conditional effect of *a3XW* on **M,** from Eq. (1), and the effect of *bM* on **Y** from Eq. (2), hence *a3bW*. For the estimation of the *index of moderated mediation*, the important part of the equation is the *a3bW* coefficient, which is a quantification of the weight of the moderator (**W**) on the indirect effect (X → M → Y). If the strength of a mediation process systematically differs as a function of the level of the moderator, then the expectations is that mediation process is conditional. In all analyses, we controlled for the effects of SES, age, gender, and immigrant background, and for the effects of family sports-socialization behaviors in relation to one other. We also controlled for T1 sports participation when looking at T2 sports participation, and T1 sports intensity when looking at T2 sports intensity. Furthermore, we inspected the collinearity diagnostics, which revealed that the variance inflation factor (VIF) values for the covariates (i.e., age, gender, immigrant background, and family socio-economic status) and predictors (i.e., role-modeling and sports-related co-activities) ranged between 1 and 1.4. In sum, the VIF values were very close to the expected value of 1, indicating lack of multicollinearity.

## Results

### Descriptive statistics and bivariate correlations

There were some significant differences across immigrant and native youth and boys and girls. For example, immigrant youth were less likely to have parents who were engaged in sports activities (a role-modeling behavior) and scored lower on the majority of the sports-participation dimensions than their native peers (see Table 1). Also, girls were more disadvantaged in terms of engaging in sports-related family co-activities, and on the majority of the sports-participation dimensions compared to the boys (see Table 2).

**Table 1:** Differences between immigrant and Nordic youth across the sports-participation dimensions and the parenting-behavior domain

	Nordic	Immigrant			
Variable	*M/%* (*SD*)	*M/%* (*SD*)	*F*/*χ*^*2*^	*p*	*η*^*2*^/ Cramer's V
Parents’ employee status	1.92 (.30)	1.29 (.78)	198.42	.001	.26
Age	14.00 (.21)	14.22 (.54)	45.06	.001	.08
Gender	53.3%	50.7%	.13	.132	.02
Role modeling	1.74 (1.30)	1.07 (1.31)	28.63	.001	.05
Sports-related co-activities	2.41 (1.14)	2.40 (1.03)	.01	.932	.00
Youth’s attainment values T1	2.66 (1.11)	2.58 (1.09)	.61	.435	.00
Youth’s attainment values T2	2.50 (1.09)	2.33 (1.01)	2.8	.094	.01
Participation in sports T1	73%	57.2%	12.97	.001	.15
Participation in sports T2	70.6%	39.6%	33.06	.001	.28
Sports intensity T1	9.31 (4.11)	8.77 (4.14)	1.09	.298	.00
Sports intensity T2	9.75 (6.00)	6.27 (5.94)	15.87	.001	.05
Continued sports participation	92.8%	71.3%	29.00	.001	.28

Partial eta-square was used as an effect-size indicator but was only estimated for the continuous and interval measures

Cramer's V effect sizes were estimated for the dichotomous variables (for which percentages rather than means and standard deviations are presented)

**Table 2:** Gender differences across the sports-participation dimensions and the parenting-behavior domain

	Girls	Boys			
Variable	*M/%* (*SD*)	*M/%* (*SD*)	*F*/*χ*^*2*^	*p*	*η*^*2*^/ Cramer's V
Parents’ employee status	1.74 (.55)	1.79 (.53)	1.53	.217	.00
Age	14.10 (.42)	14.09 (.36)	.16	.69	.00
Immigrant background	52.4%	50.7%	.13	.132	.02
Role modeling	1.57 (1.40)	1.49 (1.27)	.466	.495	.00
Sports-related co-activities	2.16 (.97)	2.63 (1.17)	29.81	.001	.05
Youth’s attainment values T1	2.47 (1.04)	2.79 (1.13)	13.43	.001	.02
Youth’s attainment values T2	2.29 (1.00)	2.59 (1.01)	12.60	.001	.02
Participation in sports T1	57.1%	77.4%	29.06	.001	.22
Participation in sports T2	55.3%	67.8%	7.68	.01	.13
Sports intensity T1	8.57 (4.24)	9.61 (3.95)	6.39	.01	.02
Sports intensity T2	7.62 (5.19)	8.94 (5.11)	4.85	.05	.02
Continued sports participation	86.6%	88.3%	.28	.596	.03

Partial eta-square was used as an effect-size indicator but was only estimated for the continuous and interval measures

Cramer's V effect sizes were estimated for the dichotomous variables (for which percentages rather than means and standard deviations are presented)

The correlation analyses showed that the study variables were associated with each other in the expected directions. For example, parents’ role-modeling behaviors and sports-related family co-activities were significantly and positively correlated with youth’s attainment values and their sports-participation behaviors (see Table 3). More specifically, youth who had experienced socialization of sports in the family setting were more likely to be involved in organized sports, to spend more time on sports activities, and to continue with those sports activities. Similarly, youth’s attainment values were significantly and positively linked to youth’s sports-participation behaviors (i.e., sports involvement, intensity of sports involvement, and continuation with sports activities). The results also revealed some notable differences between immigrant and Nordic youth, and between boys and girls. For example, being an immigrant was negatively linked to having physically active parents (role models with regard to sports), being involved in sports activities, amount of time spent on sports activities, and continuation with sports activities. As for gender, the results indicated that being a boy was positively linked to sports-related family co-activities, attainment values, and participation in sports.

**Table 3:** Correlations among the study variables

Measure	1	2	3	4	5	6	7	8	9	10	11	12	13
1. Socio-economic status	–												
2. Age	− .20***	–											
3. Gender	.05	− .02	–										
4. Immigrant background	− .51***	.28***	− .02	–									
5. Role-modeling	.15***	− .12**	− .03	− .22***	–								
6. Sports-related co-activities	.01	− .03	.22***	− .00	.20***	–							
7. Youth’s values T1	.05	.00	.15***	− .03	.18***	.46***	–						
8. Youth’s values T2	.10*	.07	.14***	− .07	.13**	.45***	.80***	–					
9. Sports participation T1	.19***	− .13**	.22***	− .15***	.16***	.26***	.54***	.49***	–				
10. Sports participation T2	.22***	− .16**	.13**	− .28***	.12***	.33***	.52***	.57***	.69***	–			
11. Sports intensity T1	.14**	− .12**	.22***	− .14**	.17***	.33***	.63***	.57***	.78***	.61***	–		
12. Sports intensity T2	.15**	− .14**	.18***	− .24***	.18***	.42***	.60***	.67***	.60***	.82***	.71***	–	
13. Continued sports participation	.08	− .13**	.03	− .28***	.18***	.12*	.18***	.30***	.^c^	1***	.19***	.71***	–

Values for dichotomous variables: gender (girls = 0, boys = 1), immigrant background (Nordic = 0, immigrant = 1), sports participation (not involved = 0, involved = 1), and continued sports participation (did not continue = 0, continued participation = 1). Time 1 (T1) and Time 2 (T2) refer to the assessment occasions

^c^Not calculated

^*^p < .05; **p < .01; ***p < .001

### Do youth’s sports’ values mediate the link between socialization behaviors in the family context and youth’s sports participation?

Initially, we performed a series of mediation analyses to test propositions from Eccles’ model. In all the analyses, we controlled for age, gender, immigrant background, SES and the relevant sports-participation measures. Further, given that the two predictors—parents’ role-modeling behaviors and sports-related family co-activities—were significantly correlated, we decided also to control for their main effects. This allowed us to observe the unique effects of sports-related family-socialization behaviors on youth’s attainment values, and youth’s choices across the sports-participation dimensions. Sports-related family co-activities predicted youth’s sports participation, intensity of sport engagement, and sports continuation through youth’s sports’ values (see Table 4). Specifically, every one unit increase in sports’ values increased youth’s sports participation by 2.27, Exp*(B*) = 2.27, 95% CI: 1.57, 3.25, and sports continuation by 1.77, Exp*(B*) = 1.77, 95% CI: 1.20, 2.59. As for parents’ role-modeling behaviors, there were no significant direct or indirect effects on youth’s sports-participation behaviors (see Table 5). In sum, the results suggest that sports-related family co-activities and youth’s sports’ values are longitudinal explicators of youth’s overall sports engagement, amount of time spent on sports, and sports continuation.

**Table 4:** The mediating effect of youth’s sports’ values on the link between sports-related family co-activities and youth’s sports-participation behaviors

				95% CI	
	*B*	*SE*	*t/z*	LL	UL
Outcome: Sports participation					
Direct effects					
Co-activities → Sports’ values	.29	.04	6.98***	.21	.37
Co-activities → Sports participation	.47	.16	2.87**	.15	.78
Sports’ values → Sports participation	.82	.19	4.36***	.45	1.18
Indirect effect					
Co-activities → Sports’ values → Sports participation	.24	.07		.12	.42
Outcome: Sports intensity					
Direct effects					
Co-activities → Sports’ values	.24	.05	4.63***	.14	.34
Co-activities → Sports intensity	.84	.24	3.47***	.36	1.31
Sports’ values → Sports intensity	1.07	.27	3.93***	.53	1.61
Indirect effect					
Co-activities → Sports’ values → Sports intensity	.25	.10		.08	.46
Outcome: Sports continuation					
Direct effects					
Co-activities → Sports’ values	.34	.04	7.64***	.25	.42
Co-activities → Sports continuation	.22	.18	1.09	-.16	.55
Sports’ values → Sports continuation	.57	.20	2.90**	.18	.95
Indirect effect					
Co-activities → Sports’ values → Sports continuation	.19	.08		.06	.38

* = *p* < .05; ** = *p* < .01; *** = *p* < .001; CI = confidence interval; LL = lower limit; UL upper limit. Unstandardized *beta* coefficients (B), 95% ordinary least squares CI for B, and 95% bootstrap CI for the indirect effects. The following variables were entered as covariates: SES, age, gender, immigrant background, and parents’ role-modeling behaviors. We also controlled for T1 sports participation when looking at T2 sports participation, and T1 sports intensity when looking at T2 sports intensity

**Table 5:** The mediating effect of youth’s sports’ values on the link between parents’ role-modeling behaviors and youth’s sports-participation behaviors

				95% CI	
	*B*	*SE*	*t*/*z*	LL	UL
Outcome: Sports participation					
Direct effects					
Role modeling → Sports’ values	.01	.03	.22	− .06	.07
Role modeling → Sports participation	.22	.12	1.89	− .01	.45
Sports’ values → Sports participation	.82	.19	4.36***	.45	1.19
Indirect effect					
Role modeling → Sports’ values → Sports participation	.01	.03		− .06	.07
Outcome: Sports intensity					
Direct effects					
Role modeling → Sports’ values	.03	.04	.66	− .05	.11
Role modeling → Sports intensity	.05	.27	.27	− .32	.42
Sports’ values → Sports intensity	1.07	.27	3.93***	.53	1.61
Indirect effect					
Role modeling → Sports’ values → Sports intensity	.03	.05		− .01	.03
Outcome: Sports continuation					
Direct effects					
Role modeling → Sports’ values	.08	.04	2.28*	.01	.17
Role modeling → Sports continuation	.11	.14	.77	− .16	.38
Sports’ values → Sports continuation	.57	.20	2.90**	.18	.95
Indirect effect					
Role modeling → Sports’ values → Sports continuation	.05	.03		.00	.12

Unstandardized *beta* coefficients (B), 95% ordinary least squares CI for B, and 95% bootstrap CI for the indirect effects. The following variables were entered as covariates: SES, age, gender, immigrant background, and sports-related family co-activities. We also controlled for T1 sports participation when looking at T2 sports participation, and T1 sports intensity when looking at T2 sports intensity

CI = confidence interval; LL = lower limit; UL upper limit

*p < .05; **p < .01; ***p < .001

### Moderating effect of immigrant background

**Fig. 1:**
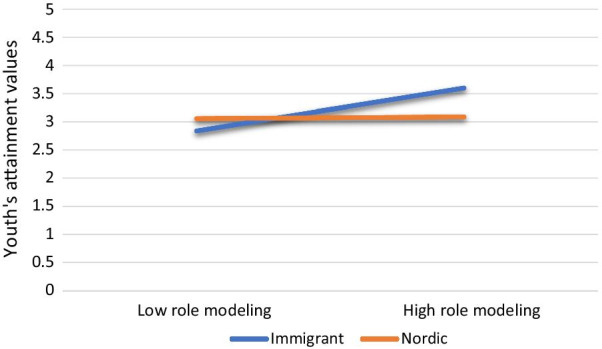
Moderating effect of immigrant background on the link between sports-related role-modeling behaviors and youth's sports-related attainment values

Next, we conducted a series of moderated mediation analyses to test whether the indirect path (i.e., family sports-socialization behaviors, through youth’s attainment values, to the sports-participation dimensions) depends on youth’s native or immigrant background. Our findings revealed that immigrant background did not moderate the mediating effect of youth’s attainment values on the association between sports-related family co-activities and the sports-participation dimensions. On the other hand, results from the moderated mediation analysis revealed that the effect of parents’ role-modeling behaviors, through youth’s attainment values, on youth’s continued sports participation, *B* = 0.17, Boot*SE* = 0.09, BootCI: 0.04, 0.38, and, intensity of sport participation, *B* = 0.33, Boot*SE* = 0.15, 95% BootCI: 0.06, 0.66, was moderated by youth’s immigrant background. More specifically, immigrant youth whose parents were engaged in physical activities were more likely to value sports activities than Nordic youth (see Fig. 1). In turn, these immigrant youth were more likely to report a high level of continued sports participation and sports intensity.

### Moderating effect of gender

Finally, we tested whether gender moderated the mediating effect of youth’s sports’ values on the link between sports-related socialization behaviors in the family context and youth’s choices across the sports-participation dimensions. Gender did not significantly moderate the mediating effect of youth’s attainment values on the link between socialization behaviors in the family context and youth’s sports-participation behaviors. Taken together, our findings suggest that the propositions from Eccles’ expectancy-value model applied equally well to boys and girls in the current sample.

## Discussion

The overall aim of the present study was to understand the associations between sports-related socialization behaviors in the family context, youth’s attainment values, and youth’s involvement in organized sport activities. More specifically, drawing on Eccles’ expectancy-value model [15] and previous research [19, 32], we tested the mediating effect of youth’s sports’ values on the link between parents’ sports-related role-modeling behaviors and family co-activities, on the one hand, and youth’s sports participation, on the other. Moreover, we examined whether the processes involving family socialization of sports, youth’s sports’ values, and youth’s involvement in organized activities are similar among immigrant and Nordic youth, and among boys and girls.

### The role of sports socialization in the family context on youth’s sports’ values and sports involvement

Consistent with previous research [19, 30], our findings indicate that family-related sports co-activities are linked to youth’s attainment values, which, in turn, help to explain youth’s sports-related behaviors one year later. According to Eccles model, the family environment plays an important role in shaping and influencing youth’s motivations in relation to specific activities [20]. More specifically, through family co-activities, family members, like parents, can convey the importance of a specific activity (e.g., sports) in various ways that, in turn, may influence youth’s valuation of and participation in that activity. For example, by engaging in sports activities with their family members, youth are implicitly socialized into the types of activities that are preferred in their family. Further, it is suggested, that, through co-activities, family members can use explicit techniques, such as verbal encouragement, to communicate the relative importance of a number of activities. Accordingly, youth can experience the extent to which certain activities (e.g., sports, music, reading) are valued in their family. Supporting these arguments, scholars have, both cross-sectionally and longitudinally, demonstrated sports-related family co-activities to be positively linked to youth’s values and activity involvement across the sporting [30, 54] and academic [19, 55] domains. In sum, through sports-related co-activities, family members have various opportunities to convey messages about the types of activities that are valued in a family, and, in turn, shape youth’s sports’ values and participation.

Contrary to the propositions derived from the expectancy-value model, parents’ role modeling was found not to predict youth’s sports-participation behaviors through youth’s attainment values. Nevertheless, our results are in line with previous studies that have found zero to marginal unique effects of parents’ role modeling (here, engagement in sports or physical activities) on youth’s sports’ values and sports involvement [30, 54, 56]. One possible explanation for this lies in how parents’ sports-related role-modeling behaviors can influence youth’s values and participation in sports activities. Eccles and colleagues [15] suggested that parents may influence children’s beliefs and behaviors through the process of observational learning. More specifically, youth may develop certain values and behaviors by observing their parents’ behaviors in relation to a specific domain, such as parental involvement in sports. Nevertheless, to understand the influence of role-modeling behaviors on youth’s beliefs and behaviors, it is equally important to consider the timing of parents’ socialization behaviors and youth’s developmental stages (e.g., in the case of childhood or adolescence, for a review see [57]. More specifically, as youth make the transition from childhood to adolescence, socializing agents other than parents (e.g., siblings, peers, or the media) may influence youth’s beliefs and behaviors. Further, as youth enter adolescence, they are likely to spend time with peers rather than their parents. Hence, parents may no longer have the same opportunities to influence their children’s beliefs and behaviors through role modeling. In line with these arguments, the studies that have found null effects of parents’ role-modeling have focused on youth in their early adolescence or have grouped together youth in their early to middle childhood and early adolescence [19, 30, 54]. Such groupings make it difficult to identify the developmental periods during which youth are relatively more susceptible to parents’ role-modeling behaviors. Hence, future studies focusing on parents’ sports-related role-modeling, as specified in the expectancy-value model, should consider youth’s developmental stages.

### The role of youth’s immigrant background

One of the main contributions of the present study is that it demonstrates that the processes involving family-socialization behaviors, youth’s attainment values, and youth’s sports participation are similar among immigrant and native youth. The current understanding, based on findings from a small number of quantitative [5] and qualitative [35] studies, along with national reports [3], is that immigrant families are less likely to be socialized into sports activities than their native counterparts. However, these findings generally indicate that there are mean-level differences between immigrant families and their native counterparts. Nevertheless, as emphasized by proponents of the no-group-differences hypothesis [58], it is important not to interpret mean-level differences across groups as an indication of different developmental processes. Specifically, it has been suggested that, despite differences across groups, the developmental processes remain similar. Supporting these arguments, scholars in the parenting literature have demonstrated that the link between parental behaviors (e.g., parental monitoring) and youth outcomes (e.g., depressive symptoms, delinquency, participation in organized sports) is similar across immigrants and their native counterparts despite mean-level differences on the factors considered [7, 59, 60]. In similar vein, despite some mean-level differences, we found that socialization behaviors in the family context, especially sports-related co-activities, are similar among both immigrant and native youth’s valuations of sports and participation in organized sports activities. Taken together, our findings extend previous research [5], and the propositions derived from the expectancy-value model [15], by demonstrating that, regardless of family background, socialization of sports in the family context plays an important role in immigrant and native youth’s sports’ values and their participation in organized sports activities over time.

Interestingly, and only for the immigrant youth, was there support for the existence of the indirect path from parents’ sports-related role-modeling behaviors, through youth’s attainment values, to youth’s continued sports involvement. A potential explanation is that immigrant youth whose parents are physically active can also be subject to other types of socialization behaviors from their parents that influence their sports’ values and continuation with sports. For example, parents who value sports activities and are physically active themselves tend to use various strategies (e.g., encouragement, spending time playing with the child, purchasing sports materials) to influence their children´s participation in the activities they value [54]. Supporting these arguments, findings from qualitative reports have revealed that immigrant parents in the U.S., especially those who have been engaged in and valued organized activities, encourage their children to participate in such activities despite transportation issues and limited financial resources [35]. In addition, parents also support their sporting offspring by attending games, even those that are far away and difficult to attend due to various barriers (e.g., transportation). Taken together, these reports indicate that immigrant parents, especially those who value organized activities, can use various forms of support to promote their children’s enrolment and continued participation in sports activities. In similar vein, our findings might be explained by the alternative strategies that immigrant parents adopt to promote their youth’s continuation in sports. Hence, future studies should examine potential similarities and differences between immigrant and native parents’ use of supportive behaviors in order to better understand why role-modeling has different implications for youth’s sports continuation across these groups.

### The role of youth’s gender

Consistent with Eccles’ expectancy-value model [15] and empirical research [19, 30], our study gives indications of sports-related gender-stereotypic behaviors in the family context. For example, boys more than girls reported spending time on sports-related family co-activities, which may reflect parents’ gender-stereotypic beliefs [15]. In fact, scholars have consistently shown that both mothers and fathers spend more time on sports-related activities with their sons than with their daughters [19, 30]. However, despite the mean-level differences, the mediation analyses revealed that sports-related family co-activities have similar implications for boys’ and girls’ sports’ values and sports behaviors. Taken together, in line with previous findings [30], and supporting proponents of the no-group-differences hypothesis [58], our findings suggest that socialization processes have similar implications for boys and girls despite the mean-level differences.

### Strengths and limitations

The findings provide practitioners, policymakers, and scholars interested in promoting youth’s participation in organized activities, such as sports, with important insights. Specifically, our findings suggest that similar family-focused interventions can be designed to facilitate immigrant and Nordic, as well as boys’ and girls’, enrolment in and continuation with organized sports activities. A second strength of the present study lies in its application of Eccles’ expectancy-value model to immigrant youth living in a Nordic country (Sweden). To our knowledge, this is the first study to apply Eccles’ model to immigrant families. Previous studies have mostly focused on European American families [18, 19, 30], or have studied immigrant families using a qualitative approach [34, 35]. Third, the present study extends previous research by examining propositions from the expectancy-value model in families with lower SES. To our knowledge, a majority of the existing studies [18–20, 30] based on Eccles’ model have relied empirically on the Childhood and Beyond (CAB) study, involving a sample of middle-class European Americans. Fourth, the measures of sports-related family co-activities support theoretical arguments from Eccles model [28], and add to the wide range of family co-activities that might be used to socialize sports activities within the family context. To our knowledge, these specific behaviors have not been tested before. Also, watching sports on TV gives practitioners and family members an affordable and doable option (also feasible for disabled family members) that can be used at home to socialize sports activities.

The present study also has some limitations that need to be acknowledged. First, we measured sports-related co-activities by asking adolescents whether they engaged in such activities with a family member. Hence, it is not possible to identify which family member (e.g., mother or father) adolescents were spending time together with in a sports context. It has been demonstrated that mothers’ and fathers’ encouragement of sports activities have different implications for their youth’s sports-related beliefs and behaviors [18, 30]. Our data are limited with regard to providing an understanding of whether mothers’ and fathers’ sports-related co-activities have similar implications for their sons’ and daughters’ sports’ values and participation. Second, we measured sports-related socialization behaviors in the family context on the basis of youth reports. There may be some discrepancies between youth reports and parents’ actual behaviors, especially for parents’ role-modeling behaviors, which were measured in terms of how often parents engaged in physical or sports activities. In order to obtain a better estimate of sports-related socialization behaviors, future studies should use both parent and youth reports. Third, even though the measures that were used in the present study are in line with theoretical arguments and previous research, some of the measures (e.g., role modeling and sports-related co-activities) were developed as part of the YeS project and needs to be validated further. Finally, we studied youth during a developmental period (adolescence) where, it is argued, that other socializing agents, such as peers, siblings and the media, as well as parents, may influence young people’s beliefs and behaviors, for a review see [57]. The simultaneous examination of different socializers may give a more comprehensive understanding of their roles in relation to each other.

### Implications of the findings and future directions

Findings from the present study offer insights into how participation in organized sports activities can be promoted among both immigrant and Nordic youth, and among boys and girls. For example, professionals working with families can be informed that, by undertaking sports activities together within the family, even just watching sports on TV, family members can shape children’s attitudes to sports and promote children’s sports participation. These findings may have important implications for policymakers and practitioners interested in promoting youth’s involvement in organized sports. This especially applies to immigrant youth, given that the literature consistently reports lower sports involvement among immigrant youth than among their native Nordic counterparts [5, 7]. Further, in light of the literature on parent’s sports-related gender-stereotypic behaviors [19, 30] and findings from the present study, scholars and practitioners interested in promoting equal gender opportunities regarding youth enrolment in and continuation with sports activities should focus on parental strategies that facilitate opportunities for girls to engage in sports-related family co-activities that are the same as those of boys. Given the fairly limited research on immigrant parents’ socialization of sports activities, future studies should examine the impact of immigrant parents’ behaviors in relation to youth sport on youth’s sports-related values and youth’s choice of activities involvement over time (e.g., continued participation vs. dropout). Given that immigrant youth are more likely to dropout from sports than their native peers [6], such an understanding would have important implications for researchers and practitioners interested in prolonging immigrant youth’s involvement in organized sports activities. Last but not least, in light of the findings the present study, we recommend future researchers to go beyond examination of mean-level differences, and investigate similarities and differences in inter-group developmental processes. This would make for a more accurate understanding of processes in the family context (e.g., links between parental behaviors and youth outcomes) across immigrant and native families.

## Conclusions

Supporting arguments from the expectancy-value model [15, 20, 28], our findings show that the socialization of sports activities in the family context plays an important role in youth’s sports-related values, which, in turn predict youth’s participation in and continuation with organized sports activities. The main contribution of the present study is that most of its findings demonstrate that the processes involving family-socialization behaviors, youth’s attainment values, and youth’s sports-participation behaviors are the same among immigrant and Nordic youth, and among girls and boys. More specifically, our findings demonstrate that immigrant and Nordic families can use similar socialization behaviors, especially family co-activities, to influence their children’s, both boys’ and girls’, sports’ values and sports involvement.

## Abbreviations

T1: Data collected during the first year of the study
T2: Data collected during the second year of the study

## Availability of data and material

The dataset generated and analyzed during the current study is not publicly available due to its longitudinal nature and the sensitivity of the questions, but is available from the corresponding author on reasonable request.
